# IL-18 But Not IL-1 Signaling Is Pivotal for the Initiation of Liver Injury in Murine Non-Alcoholic Fatty Liver Disease

**DOI:** 10.3390/ijms21228602

**Published:** 2020-11-14

**Authors:** Simon Hohenester, Veronika Kanitz, Tobias Schiergens, Claudia Einer, Jutta Nagel, Ralf Wimmer, Florian P. Reiter, Alexander L. Gerbes, Enrico N. De Toni, Christian Bauer, Lesca Holdt, Doris Mayr, Christian Rust, Max Schnurr, Hans Zischka, Andreas Geier, Gerald Denk

**Affiliations:** 1 Department of Medicine II, University Hospital, LMU Munich, 81377 Munich, Germany; jutta.nagel@med.uni-muenchen.de (J.N.); ralf.wimmer@med.uni-muenchen.de (R.W.); florian.reiter@med.uni-muenchen.de (F.P.R.); gerbes@med.uni-muenchen.de (A.L.G.); Enrico.deToni@med.uni-muenchen.de (E.N.D.T.); gerald.denk@med.uni-muenchen.de (G.D.); 2 Institute of Pathology, Faculty of Medicine, LMU Munich, 80337 Munich, Germany; veronika.kanitz@med.uni-muenchen.de (V.K.); Doris.Mayr@med.uni-muenchen.de (D.M.); 3 Department of General, Visceral and Transplantation Surgery, University Hospital, LMU Munich, 81377 Munich, Germany; tobias.schiergens@med.uni-muenchen.de; 4 Institute of Molecular Toxicology and Pharmacology, Helmholtz Center Munich, German Research Center for Environmental Health, 85764 Neuherberg, Germany; claudia.einer@helmholtz-muenchen.de (C.E.); zischka@helmholtz-muenchen.de (H.Z.); 5 Division of Gastroenterology, Endocrinology, Infectiology and Metabolism, University Hospital Giessen and Marburg, Campus Marburg, Philipps University Marburg, 35043 Marburg, Germany; chr.bauer@uni-marburg.de; 6 Institute of Laboratory Medicine, University Hospital, LMU Munich, 81377 Munich, Germany; Lesca.Holdt@med.uni-muenchen.de; 7 Department of Medicine I, Hospital Barmherzige Brüder, 80639 Munich, Germany; christian.rust@barmherzige-muenchen.de; 8 Division of Clinical Pharmacology, University Hospital, LMU Munich, 80336 Munich, Germany; max.schnurr@med.uni-muenchen.de; 9 Institute of Toxicology and Environmental Hygiene, Technical University Munich, School of Medicine, 80802 Munich, Germany; 10 Division of Hepatology, University Hospital Würzburg, 97080 Würzburg, Germany; Geier_A2@ukw.de; 11 Transplantation Center Munich, University Hospital, LMU Munich, 81377 Munich, Germany

**Keywords:** NAFLD, Western diet, NLRP3, inflammasome, interleukin 1, interleukin 18, NASH, ALiOS

## Abstract

Non-alcoholic fatty liver disease (NAFLD) is rising in prevalence, and a better pathophysiologic understanding of the transition to its inflammatory phenotype (NASH) is key to the development of effective therapies. To evaluate the contribution of the NLRP3 inflammasome and its downstream effectors IL-1 and IL-18 in this process, we applied the true-to-life “American lifestyle-induced obesity syndrome” (ALiOS) diet mouse model. Development of obesity, fatty liver and liver damage was investigated in mice fed for 24 weeks according to the ALiOS protocol. Lipidomic changes in mouse livers were compared to human NAFLD samples. Receptor knockout mice for IL-1 and IL-18 were used to dissect the impact of downstream signals of inflammasome activity on the development of NAFLD. The ALiOS diet induced obesity and liver steatosis. The lipidomic changes closely mimicked changes in human NAFLD. A pro-inflammatory gene expression pattern in liver tissue and increased serum liver transaminases indicated early liver damage in the absence of histological evidence of NASH. Mechanistically, *Il-18r^−/−^*- but not *Il-1r^−/−^* mice were protected from early liver damage, possibly due to silencing of the pro-inflammatory gene expression pattern. Our study identified NLRP3 activation and IL-18R-dependent signaling as potential modulators of early liver damage in NAFLD, preceding development of histologic NASH.

## 1. Introduction

Non-alcoholic fatty liver disease (NAFLD), the hepatic manifestation of the metabolic syndrome, has become a leading cause of chronic liver disease in many regions of the world [1], is still on the rise [2,3] and has been termed the “first pandemic” of the 21st century [4]. In absence of established pharmacological treatment options, the current state-of-the-art therapy is lifestyle intervention [5], which could be shown effective even in advanced, morbid obesity [6].

Various risk factors have been associated with the development of NAFLD, including dietary habits, lifestyle, gut microbiome and drug intake [7,8,9,10,11]. NAFLD comprises a wide spectrum from simple hepatic steatosis to non-alcoholic steatohepatitis (NASH), characterized by (necro)inflammation and fibrosis that develop in 20% of patients with NASH. Many of these patients eventually progress to cirrhosis and/or hepatocellular carcinoma [4,5].

Factors responsible for the transition of benign steatosis towards NASH remain enigmatic but may involve, among others, mitochondrial dysfunction [12,13,14]. In humans, a detailed description of the fatty acid composition in NASH livers has been established [15]. Fatty acid composition itself is thought to influence the development of NASH, possibly via the NLRP3 [16] inflammasome. Inflammasomes are intracellular multiprotein complexes that sense endogenous and exogenous danger signals through NOD-like receptors (NLRs) [17,18]. Activation of sensors of intracellular inflammasome complexes, such as the PYD domain-containing protein 3 (NLRP3), together with the adaptor molecule ASC, leads to activation, hence cleavage of pro-caspase-1 to activated caspase-1 [17]. This promotes activation of the pro-inflammatory cytokines interleukin-1β (IL-1β) and IL-18, resulting in a sustained inflammatory response [19]. 

It has been shown that the methionine–choline-deficient (MCD) diet, a widely used in vivo model of NASH in mice, induces upregulation of the NLRP3 inflammasome, and that high-fat diet (HFD) feeding can also cause inflammasome activation [20]. Investigation of the pathogenesis of NAFLD and factors contributing to the progression to NASH has strongly relied on these two types of mouse models. Their common drawback is their utmost artificial nature. In MCD-fed mice, NASH develops in the absence of obesity or insulin resistance. Even more, the animals lose weight during treatment, hardly reflecting the pathogenesis of NASH in most affected patients. Commonly applied HFD models, on the other hand, utilize a diet composition that is likewise highly artificial, and transferability of results gained in these animals might be limited [21]. 

Therefore, we applied the American lifestyle-induced obesity syndrome (ALiOS) model, a true-to-life mouse model of obesity. This diet reflects commonly consumed fast food. Chow provides 45% of calories from fat, including trans fats, and high-fructose corn syrup (HFCS, 55% fructose, 45% glucose by weight, 42 g/L) is administered in drinking water as an equivalent for soda drinks. This diet has been shown to induce obesity, glucose intolerance, hyperinsulinemia and substantial hepatic steatosis associated with necroinflammation and a profibrogenic response in mice [22].

Applying this model, we revisited the contribution of the NLRP3 inflammasome, i.e., its effector cytokines IL-1 and IL-18, for the development of liver damage in NAFLD. To block IL-1- and IL-18-dependent signaling, knockout animals for the respective receptors were used.

## 2. Results

### 2.1. Mice Fed ALiOS Diet Develop Obesity, Metabolic Syndrome and Fatty Liver

**Figure 1:**
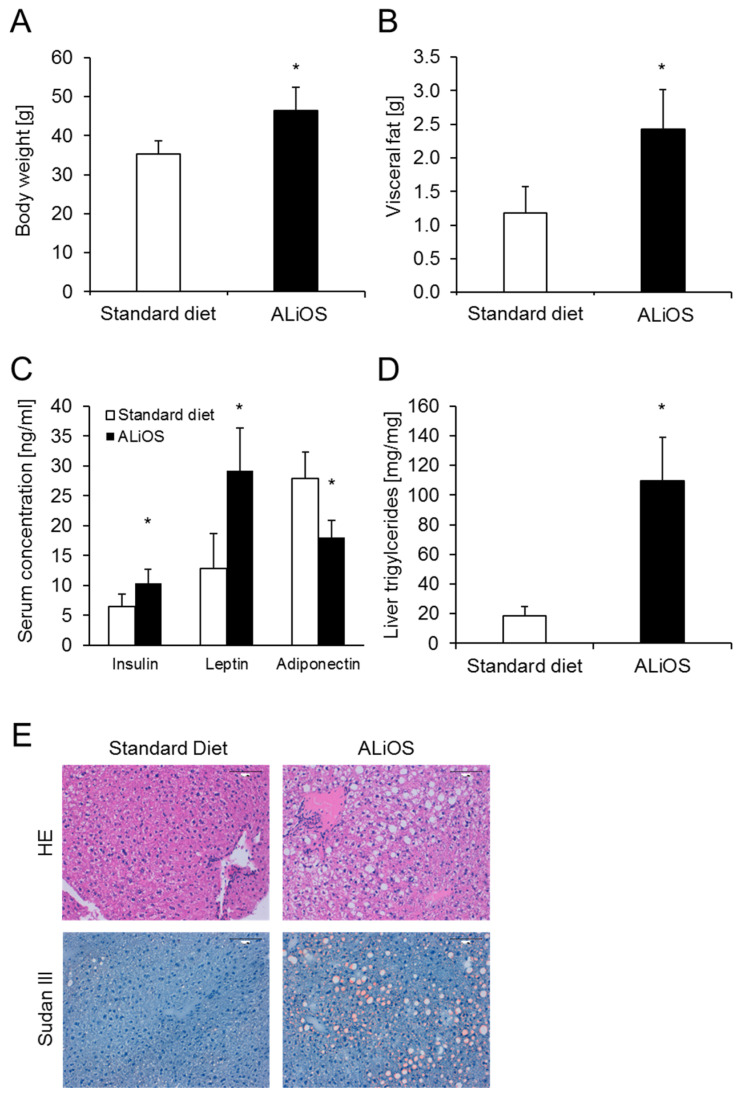
American lifestyle-induced obesity syndrome (ALiOS) diet in mice induces obesity, metabolic syndrome and fatty liver. Wildtype mice were fed standard diet or ALiOS diet for 24 weeks. (**A**) Body weight (*n* = 14, each), (**B**) visceral fat weight (*n* = 8, each), (**C**) serum concentrations of insulin, leptin and adiponectin (*n* = 8, each, 12–24 weeks combined) and (**D**) liver triglycerides (*n* = 4, each) at the end of the study are shown as mean ± standard deviation (* *p* < 0.01, t-test). (**E**) Representative liver sections following H&E and Sudan red staining showing micro- and macrovesicular steatosis and heterogenicity in cell and nuclear size but a lack of ballooning or an inflammatory infiltrate (200×, bar represents 100 μM).

Wildtype C57BL/6 mice were fed a Western diet according to the ALiOS protocol, or control diet, for 24 weeks. ALiOS-fed mice had an increase in total body weight of 32% compared to standard diet-fed mice (Figure 1A), and especially an increase of visceral fat (Figure 1B). ALiOS feeding resulted in metabolic syndrome, as indicated by an increase in serum insulin and leptin levels and a drop of adiponectin levels (Figure 1C). The decrease in systemic adiponectin levels was accompanied by reduced adiponectin mRNA expression in visceral fat. By contrast, expression of leptin, F4/80, IL-6, TNF, MCP1 and IL-1 in visceral fat was unchanged (not shown).

Notably, marked hepatic accumulation of triglycerides was found (Figure 1D) and livers stained strongly positive for lipid droplets (Figure 1E). In the histopathologic evaluation of H&E and Sudan red stains, micro- and macrovesicular steatosis and heterogenicity in cell and nuclear size were seen (Figure 1E). 

Thus, ALiOS-fed mice developed obesity, features of metabolic syndrome and hepatic steatosis.

### 2.2. The Hepatic Lipidome in the ALiOS Model Closely Mimics Changes in the Human Hepatic Lipidome in NAFLD

**Figure 2:**
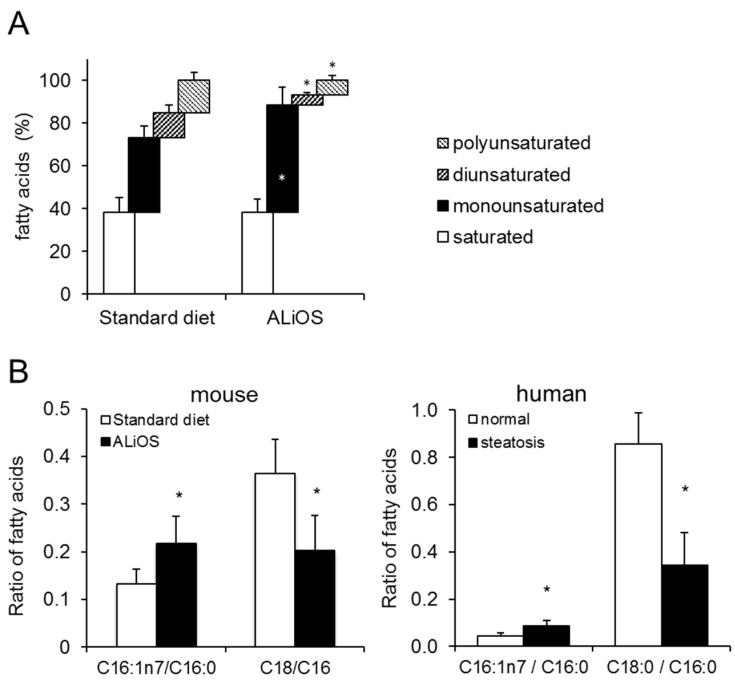
Comparison of the liver lipidome in murine and human NAFLD. (**A**) Wildtype mice were fed standard diet or ALiOS diet for 24 weeks. Liver lipidome was characterized by gas chromatography (GC) as described. Composition of the lipidome is depicted in categories of saturated, monounsaturated, diunsaturated and polyunsaturated fatty acids. (**B**) Fatty acid composition of mouse and human livers without steatosis (white bars) and with steatosis (black bars) was determined. Previously described fatty acid ratios associated with NAFLD are depicted (mean ± standard deviation; *n* = 5 for mouse, *n* = 9–10 for human liver tissue; * *p* < 0.05; t-test).

Feeding of the ALiOS diet led to marked changes in the hepatic fatty acid composition. While the proportion of monounsaturated fatty acids increased, both di- and polyunsaturated fatty acids decreased in liver tissue (Figure 2A). Moreover, while the C16:1n7/C16 ratio was increased in ALiOS diet-fed animals, the C18:0/C16:0 ratio dropped (Figure 2B). Next, we determined the fatty acid composition in livers from human samples of NAFLD. Importantly, specific fatty acid indices were similarly altered in human liver and steatotic mouse samples alike (Figure 2B). A more detailed analysis of fatty acid species detectable in ALiOS diet-fed animals is shown in Supplementary Figure S1A.

### 2.3. ALiOS Diet Leads to Activation of the Inflammasome

**Figure 3:**
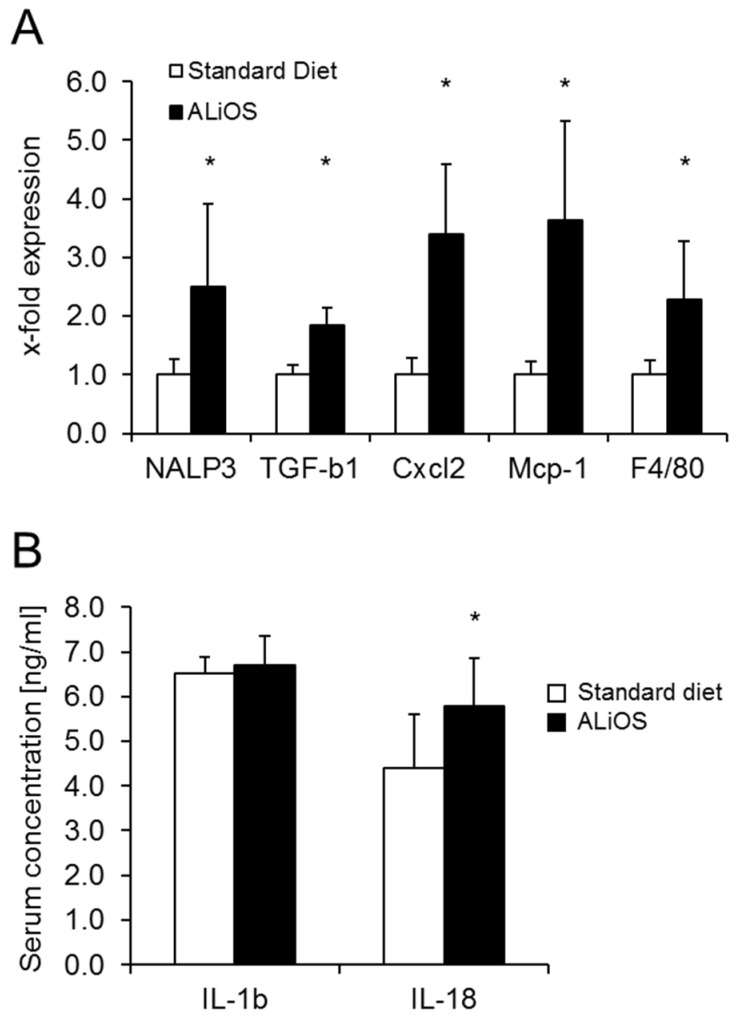
Engagement of the inflammasome pathway following ALiOS diet Wildtype mice were fed standard diet (white bars) or ALiOS diet (black bars) for 24 weeks. (**A**) Expression of indicated genes in liver tissue and (**B**) serum concentrations of IL-1β and IL-18 (*n* = 8, each) at the end of the study are shown (mean ± standard deviation; *n* = 6, each; * *p* < 0.01, t-test).

ALiOS feeding was associated with an upregulation of NLRP3, indicative of activation of this signaling pathway (Figure 3A). Along with NLRP3 activation, expression of the pro-inflammatory genes TGF-β1, Cxcl2, Mcp1 and F4/80 was increased, indicating an active inflammatory environment in liver tissue.

Activation of the inflammasome was confirmed by measurement of serum levels of NLRP3-associated cytokines. Serum levels of IL-1β were unaltered. In contrast, serum levels of IL-18 were increased (Figure 3B). IL-1α levels were comparatively low and without a significant difference (182 ± 145 vs. 125 ± 48 pg/mL in control vs. ALiOS diet, *n* = 7, each). Likewise, serum levels of IL-10, MCP and TNF-α were not different in both experimental groups. 

In summary, the ALiOS diet resulted in an activation of the inflammasome and was associated with an upregulation of IL-18 as well as pro-inflammatory markers in the liver.

### 2.4. ALiOS Diet Leads to NAFLD but Not NASH

**Figure 4:**
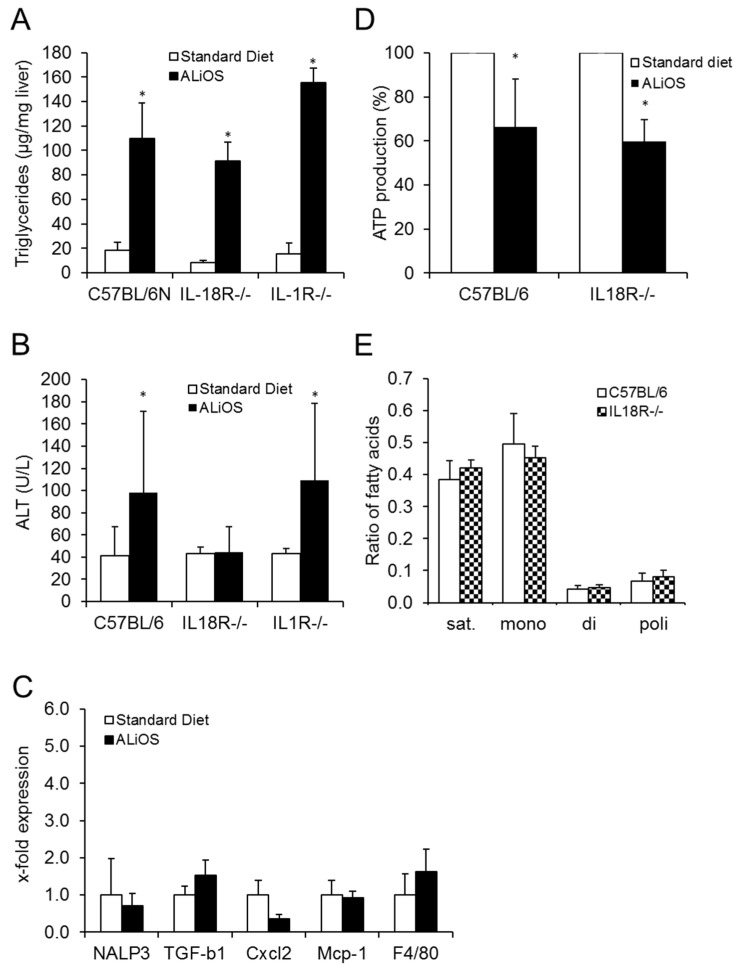
IL-18R- but not IL-1R-dependent signaling promotes liver damage in ALiOS diet-induced NAFLD. Mice were fed standard diet (white bars) or ALiOS diet (black bars) for 24 weeks. (**A**) Liver triglycerides (*n* = 4, each; * *p* < 0.01; t-test) and (**B**) serum ALT levels (*n* = 8–14; * *p* < 0.05; *t*-test) are shown for indicated genotypes as mean ± standard deviation. (**C**) Depiction of gene expression in liver tissue of standard diet- (white bars) and ALiOS diet-fed *Il-18r ^−/−^* mice (*n* = 6, each; * *p* < 0.01, *t*-test). (**D**) ATP productivity was determined in mitochondria isolated from liver tissue following 24 weeks of standard diet (white bars) and ALiOS diet (black bars) in wildtype and *Il-18r ^−/−^* mice. (E) Ratios of major hepatic lipidome components (saturated, monounsaturated, diunsaturated and polyunsaturated fatty acids) in ALiOS diet-fed wildtype (black bars) and *Il-18r ^−/−^* (chess field) mice (*n* = 5, each, no statistical difference).

In wildtype animals, ALiOS feeding led to mild but chronic hepatocellular damage. This was evidenced by increased serum activities of ALT, indicating hepatopathy associated with NAFLD in these animals (Figure 4B). Despite such liver damage and despite the increased expression of pro-inflammatory TGF-β1, Cxcl2, Mcp1 and F4/80, no distinct inflammatory infiltrate was detected on histology. Applying the established histological scores SAF (steatosis, activity, fibrosis), NAS (NAFLD Activity Score) and the rodent NASH score in a blinded fashion, no evidence of NASH was detected (Table 1). Accordingly, measurement of liver hydroxyproline showed the absence of advanced liver fibrosis (Table 2). 

Taken together, feeding of the ALiOS diet without simultaneous restrictions of voluntary movement induced obesity, metabolic syndrome and NAFLD in C57BL/6 wildtype mice, but not NASH.

### 2.5. IL-18 but Not IL-1 Is a Downstream Mediator of the Activated Inflammasome in NAFLD

To delineate the role of IL-18 and IL-1 in inflammasome-mediated signaling in early NAFLD, we blocked these signaling pathways using *Il-18r ^−/−^* and *Il-1r ^−/−^* mice. Body weight, visceral fat weight and serum non-esterized fatty acids (NEFA) were not different between the genotypes (Table 2). No differences in the degree of liver steatosis were detected when comparing liver triglyceride levels. After ALiOS diet feeding for 24 weeks, triglyceride levels were increased by 5.6-, 10.9- and 10.1-fold in wildtype, *Il-18r ^−/−^* and *Il-1r ^−/−^* mice, respectively (Figure 4A). Interestingly, however, *Il-18r ^−/−^* but not *Il-1r ^−/−^* animals were protected against steatotic liver injury (Figure 4B). 

Thus, while the knockout of IL-18R or IL-1R did not influence the development of obesity and liver steatosis, NAFLD-associated hepatocellular damage as reflected by ALT elevation was abrogated in *Il-18r ^−/−^* animals.

### 2.6. IL-18R Deficiency Leads to Silencing of the Pro-Inflammatory Environment in Steatotic Livers

Downstream of the activation of the inflammasome, the pro-inflammatory gene expression profile that had developed upon ALiOS diet feeding in wildtype animals was abrogated in *Il-18r ^−/−^* mice (Figure 4C). Typically, mitochondrial dysfunction is considered to occur upstream of inflammasome activation, but activation of the inflammasome may also impact mitochondrial function. As depicted in Figure 4D, ATP production capacity in mitochondria isolated from mouse livers was diminished in NAFLD but this result was unaltered in *Il-18r ^−/−^* mice. Moreover, hepatic fatty acid composition remained unaffected by the animals’ genotype (Figure 4E and Supplementary Figure S1B).

Thus, in *Il-18r ^−/−^* animals, activation of the inflammasome in steatotic livers was abrogated and the development of a pro-inflammatory environment in the liver was prevented independently of fatty acid composition or mitochondrial dysfunction in hepatic steatosis.

## 3. Discussion

The prevalence of obesity and metabolic syndrome is increasing [1,2,3]. The exact factors, however, mediating the transition of non-alcoholic fatty liver disease to non-alcoholic steatohepatitis are still not fully determined. The data derived from this current study indicate that NLRP3 activation and IL-18R- but not IL-1R-dependent signaling may be potential modulators of early liver damage in NAFLD.

The MCD and HFD mouse models of NASH inadequately reflect changes seen in humans, both with regard to metabolic syndrome and fatty acid composition in the liver. Here, we utilized the ALiOS diet model, which very closely resembles dietary habits in the Western world, and demonstrate that it induces obesity, metabolic syndrome and NAFLD in C57BL/6 mice (Figure 1). This diet has previously been shown to induce obesity, glucose intolerance, hyperinsulinemia and substantial hepatic steatosis associated with necroinflammation and a profibrogenic response in mice [22]. Therefore, a detailed lipidomic analysis was performed in mouse liver tissue. Analyzing the lipidome in liver tissue from our mice, we found that changes in specific fatty acid ratios very closely resembled lipidomic changes in our human NAFLD samples (Figure 2B). Similar changes in human NAFLD have previously been reported [15], validating our results. Hence, our results further underscore the true-to-life nature of the ALiOS model, reflecting both metabolic syndrome and lipidomic changes of human NAFLD. In contrast to the initial description of the ALiOS model, no necroinflammation, i.e., NASH, was found under our experimental conditions [22]. This was most likely due to the fact that the sedentary lifestyle described herein was not reflected in our setting. 

NAFLD in our model was, however, accompanied by a pro-inflammatory hepatic gene expression pattern and activation of the inflammasome (Figure 3). It has been shown that MCD diet and high-fat diet (HFD) feeding induces upregulation of the NLRP3 inflammasome, and can also cause inflammasome activation [20]. In humans, both cellular and humoral changes occur during early steatosis without overt pathological signs of NASH [23]. Saturated fatty acids can upregulate and activate the inflammasome complex in hepatocytes and induce IL-1ß production [20]. Preliminary data from the Würzburg cohort of NAFLD patients indicate a correlation between the genetic variant rs10754558 in the *NLRP3* inflammasome and elevated ALT levels, suggesting a role of this inflammasome in early steatotic liver injury (Geier A. et al., unpublished data).

In our animals, NLRP3 was upregulated, as well as TGF-ß1, a major pro-fibrogenic cytokine, and CXCL2. CXCL2 is produced in reaction to lipopolysaccharides by macrophages or monocytes. Monocyte markers Mcp-1 and F4/80 were also increased, indicating an inflammatory process. This is reflected by the increase in ALT.

Portal macrophages are detectable in steatosis alone as the earliest change, followed by an elevated expression of pro-inflammatory cytokines such as IL-1α and TNF-alpha in early NASH. In our study, the downstream mediator of NLRP3, IL-18 but not IL-1, was particularly increased. In mice on a high-fat diet, IL-18 has previously been identified among the intermediate markers of hepatic steatosis [24] and similar findings have been obtained in early steatosis in rabbits [25]. In humans, elevated serum IL-18 concentration correlated with ALT as a marker of hepatocyte injury in obese children [26]. Accordingly, IL-18 has been considered to play a role in predicting advanced liver steatosis and fatty liver in obese children. This prompted us to further explore the significance of this cytokine in early NAFLD.

Knockout of IL-18R did not influence the development obesity and markers of metabolic syndrome (Table 2). In contrast, it completely abrogated the increase in pro-inflammatory gene expression both in the liver and in serum ALT, a systemic marker for liver damage (Figure 4). The insignificance of IL-18 for the development of metabolic syndrome is in line with a study in humans where an anti-IL-18 monoclonal antibody was ineffective for the treatment of type 2 diabetes mellitus [27]. Unfortunately, this study did not investigate the hepatic phenotype of these patients. Interestingly, our findings are in contrast to previous results in the MCD diet mouse model where NASH severity was exacerbated in IL-18 knockout mice [28], pointing out a context-dependent role for IL-18.

In contrast to IL-18R, the IL-1R knockout was without benefit in the ALiOS model and liver damage was not different from wildtype animals. This is in line with previous work on cholestatic liver disease, where IL-1 signaling also was of limited importance [29] but in contrast to the choline-deficient mouse model where NASH was ameliorated by the IL-1R knockout [30]. In a model of hypercholesterinemia, lack of IL-1α and IL-1β was able to prevent liver inflammation [31]. However, in this model, liver inflammation developed despite a decrease in liver triglycerides in this model, again reflecting a rather artificial setting. Taken together, the effects of IL-1R-dependent signaling, too, may be context-dependent.

The activation of the NLRP3 inflammasome is upstream of both IL-1 and IL-18 signaling. It has previously been reported that the knockout of this upstream target did not prevent steatosis-associated liver damage [12,13]. Mitochondrial dysfunction is an early event in the development of steatotic liver damage [12,13], and is considered to occur upstream of inflammasome activation. Vice versa, activation of the inflammasome may also impact mitochondrial function [32], and the specific sequence of events in NALFD is unknown. In mitochondria isolated from livers from ALiOS-fed animals, ATP production capacity was markedly diminished compared to controls, indicating mitochondrial dysfunction (Figure 4D). This was unaltered by IL-18R deficiency. This supports the view that mitochondrial dysfunction is not dependent on inflammasome activity and IL-18 signaling.

The major limitation of this study is the absence of NASH upon histopathologic evaluation. Our study thus focuses on the very early changes in steatotic liver and it remains to be determined whether IL-18R-dependent signaling also drives inflammation in established NASH. Since the true-to-life nature of the experimental model applied in this study is one of its strengths, investigation of longer time periods of feeding or introduction of an additional hit, rather than applying more artificial models such as the MCD diet, may be suitable to further explore these questions.

In summary, we provide evidence that the ALiOS mouse model used in this study particularly closely mimics the pathophysiology of human NAFLD, including obesity, liver steatosis and lipidomic changes. We were able to identify IL-18-dependent signaling as a modulator of early liver damage in fatty liver, preceding development of histologic NASH. Further studies will have to elucidate to what extent IL-18R deficiency is protective in non-alcoholic steatohepatitis.

## 4. Materials and Methods

### 4.1. Animal Experiments

Male C57BL/6NCrl mice from Charles River (Karlsruhe, Germany) and Il-18r ^−/−^ and Il-1r ^−/−^ mice were co-housed and kept according to the guidelines for the care and use of laboratory animals at the University Hospital Munich. Mice had free access to water. Water for ALiOS diet-fed animals was enriched with 23.1 g/L fructose and 18.9 g/L glucose reflecting consumption of soda drinks in humans. Animals were either fed with the ALiOS (Altromin, Lage, Germany) or with the standard diet (ND, Ssniff V1534, Soest, Germany) for 24 weeks starting at the age of eight weeks. A total of 45% of the calories of the ALiOS diet originated from fat (control: 9% from fat) in the form of partially hydrogenated vegetable oil (28% saturated fatty acids, 57% monounsaturated fatty acids (MUFAs) and 13% polyunsaturated fatty acids (PUFAs) [22]. Animal experiments were approved by local authorities (55.2-1-54-2531.65.10; 15 November 2010).

### 4.2. Human Tissue

Human liver tissue was obtained from the Biobank of the Department of General, Visceral and Transplantation Surgery, Ludwig Maximilian University, Munich, Germany, under the administration of the Human Tissue and Cell Research Foundation (HTCR) [21], as approved by the local ethics committee (025–12).

### 4.3. Serum Biochemistry

Activity of alanine aminotransferase was quantified from fresh serum by a respons^®^ 910 fully automated analyzer (DiaSys, Holzheim, Germany).

### 4.4. Liver Histology and Determination of Triglycerides and Hydroxyproline in Liver Tissue

Liver tissue was embedded in paraffin after 24-h fixation in 4% formaldehyde solution or was shock-frozen in liquid nitrogen. Paraffinized blocks were cut into slices of 4 μm thickness that were mounted on SuperFrost Plus microscopic slides (Menzel Gläser, Darmstadt, Germany). After stepwise deparaffinization and rehydration, the slides were stained with hematoxylin and eosin (H&E). Staining of shock-frozen tissue with Sudan III and H&E was performed according to standard protocols. 

To establish the diagnosis of NASH, different well-established scoring systems were used. Both the SAF score (encompassing an assessment of steatosis, activity and fibrosis) [33] and the NAS score (NAFLD activity score) [34] were applied, as well as the rodent NASH score [35], which was specifically evaluated for use in animal models.

For quantification of triglycerides (TGs) in liver, 100 mg of liver tissue was sonicated in 1 mL 5% NP40 solution, heated for 5 min at 96 °C and cooled down on ice. Afterwards, the homogenate was centrifuged for 2 min at 20,000× *g* and the supernatant was analyzed with a Response 910 system (DiaSys, Holzheim, Germany).

Hydroxyproline levels in liver tissue, serving as a read-out for liver fibrosis, were quantified according to Edwards et al. [36]. In brief, liver tissue was homogenized with a 20-fold volume of hydrochloric acid (6 mol/L). After hydrolyzation overnight at 110 °C, filtrates were neutralized with NaOH 2 mol/L and oxidized with chloramin T. Surplus of chloramin T was degraded with perchloric acid and the sample was derivated with 4-(dimethylamino)benzaldehyde at 60 °C for 20 min. Absorption was measured at a wavelength of 565 nm. Concentration was calculated by comparison to a standard curve.

### 4.5. Analysis of Fatty Acids by Gas Chromatography

Fatty acids were extracted from mouse tissue (10 mg) according to the method of Folch [37]. Cleared extracts were washed with 0.9% NaCl and twice with methanol/water (1:1 *v*/*v*). The organic phase was evaporated (N2), esterified and extracted with n-hexane. Fatty acid methyl esters were separated by capillary gas chromatography with a BPX70 column from SGE at an oven temperature of 120–210 °C with an increase of 2 °C/min, H2 as the carrier gas and a flame ionization detector, and were identified by their retention time relative to standard mixtures (37 component FAME mix, Supelco, Taufkirchen, Germany). Quantification was performed with C15:0 as the internal standard and with Clarity Lite software (DataApex, Prague, Czech Republic). For comparison with mouse livers from our experiments, anonymized samples of human liver tissue from the Biobank of the Department of General, Visceral and Transplantation Surgery, Ludwig Maximilian University, Munich, Germany, under the administration of the HTCR [38], were evaluated for liver steatosis and inflammation on histology and the lipidome was determined as described above for mouse tissue.

### 4.6. Isolation of Mitochondria

Mitochondria from mouse livers were freshly prepared and purified by Percoll™ (GE Healthcare, Freiburg, Germany) as described [39]. ATP synthesis capacity was analyzed by the ATP Bioluminescence Assay Kit (Roche, Penzberg, Germany).

### 4.7. PCR

mRNA was isolated from 30 mg liver tissue using Trizol reagent (VWR, Ismaning, Germany) according to the manufacturer’s instructions. Visceral fat (50 mg) was minced with a Polytron homogenizer (Kinematica, Eschbach, Germany) and mRNA was extracted with NucleoSpin^®^ RNA kits (Machery-Nagel, Düren, Germany) according to the manufacturer’s instructions. Complementary DNA was synthesized using reverse transcriptase SuperScript^®^ III (Invitrogen, Carlsbad, CA, USA). Real-time PCR was performed in a SYBR^®^ Green system (QuantiTect SYBR Green PCR Kit, Qiagen, Venlo, The Netherlands) on a Realplex^4^ Mastercycler epgradient S (Eppendorf, Eppendorf, Germany). Expression was calculated according to the ΔΔCt method with GAPDH and 36B4 as the housekeeping genes and was normalized to the means of the controls. Primers were purchased at Eurofins Genomics (Ebersberg, Germany), and the sequences were as follows:
Gapdh:leftAGCGAGACCCCACTAACATCrightGGCGGAGATGATGACCCTTT36B4:leftTCTAGGACCCGAGAAGACCTrightCCCACCTTGTCTCCAGTCTTNalp3:leftAAAGCTAAGAAGGACCAGCCrightTATCCCAGCAAACCCATCCATgf-b1:leftCGCAACAACGCCATCTATGArightACTGCTTCCCGAATGTCTGACxCl2:leftTCTTGAGCTTGGTGACAAAAACrightGGCTGGAGAGCTACAAGAGGMcp-1:leftGCTGCTACTCATTCACCAGCrightCTTCTTGGGGTCAGCACAGAF4/80:leftAACCAACTTTCAAGGCCCAGrightTGCAGACTGAGTTAGGACCA

### 4.8. Multiplex Assay

Interleukin (IL)-1α, IL-1β, IL-6, IL-10 and IL-18, insulin, leptin and adiponectin were quantified in serum with the customized Bio-Plex Pro™ Cytokine and Diabetes Assay and the Bio-Plex 200 system from Bio-Rad according to the manufacturer’s instructions.

### 4.9. Statistical Testing

Results from independent repeated experiments are shown as means ± standard deviation. The Shapiro–Wilk test was used to demonstrate normal distribution of fraction samples. Differences between means of two groups were validated with the t-test. Statistical testing of observed frequencies was performed by the chi-squared test or Fisher’s exact test, as appropriate. All statistic calculations were performed with the SPSS 25 software package (IBM).

## Figures and Tables

**Table 1:** Histological scoring for fatty liver disease. Wildtype mice were fed standard diet or ALiOS diet for 24 weeks. Liver sections were categorized by a blinded pathologist according to the indicated scoring systems. *p* value for the Fisher test is given.

**Rodent Score**	**No NAFLD**	**NAFL**	**NASH**	***p***
Standard Diet	4	2	0	
ALiOS	0	6	0	0.061
**NAS**	**No NAFLD**	**NAFL**	**NASH**	
Standard Diet	6	0	0	
ALiOS	0	6	0	**0.002**
**SAF**	**No NAFLD**	**mild disease**	**Moderate or severe**	
Standard Diet	6	0	0	
ALiOS	0	6	0	**0.002**

**Table 2:** Effect of genotype on body composition, serum fatty acids and liver fibrosis. Wildtype mice were fed standard diet or ALiOS diet for 24 weeks. Total values (mean ± standard deviation) and fold increase are given for body weight, visceral fat, non-esterized fatty acids (NEFA) in serum and hydroxyproline in liver tissue (*n* = 14 for wildtype, *n* = 8 for IL-18R^−/−^, *n* = 10 for IL-1R^−/−^; ** *p* < 0.01 compared to standard diet, *t*-test).

	C57BL/6 wt MW ± SD (-Fold Increase)	IL18R^−/−^ MW ± SD (-Fold Increase)	IL1R^−/−^ MW ± SD (-Fold Increase)
**Body weight (g)**			
Standard diet	35.22 ± 3.37	32.72 ± 2.17	34.87 ± 2.84
ALiOS	46.44 ± 5.97 **	38.82 ± 3.28 **	45.28 ± 2.09 **
	(1.32)	(1.19)	(1.21)
**Visceral fat (g)**			
Standard diet	1.18 ± 0.40	0.54 ± 0.10	1.21 ± 0.36
ALiOS	2.43 ± 0.59 **	1.85 ± 0.59 **	2.48 ± 0.22 **
	(2.06)	(3.43)	(2.05)
**NEFA (mmol/L)**			
Standard diet	0.46 ± 0.11	0.53 ± 0.11	0.72 ± 0.20
ALiOS	0.93 ± 0.23 **	1.10 ± 0.21 **	0.98 ± 0.14 **
	(2.02)	(2.08)	(1.36)
**Hydroxyprolin (µg/g)**			
Standard diet	84.91 ± 44.15	137.39 ± 50.37	117.08 ± 52.64
ALiOS	115.66 ± 77.12	122.78 ± 35.86	96.25 ± 34.55
	(1.36)	(0.89)	(0.82)

## Supplementary Materials

Supplementary materials can be found at https://www.mdpi.com/1422-0067/21/22/8602/s1.
