# Supplementary material for: IL-18 But Not IL-1 Signaling Is Pivotal for the Initiation of Liver Injury in Murine Non-Alcoholic Fatty Liver Disease
Source: Int J Mol Sci. 2020 Nov 14;21(22):8602. doi: 10.3390/ijms21228602 (PMC7696705; doi:10.3390/ijms21228602)
Supplement: Supplementary file 1 [file ijms-21-08602-s001.pdf]

Supplementary Figure 1

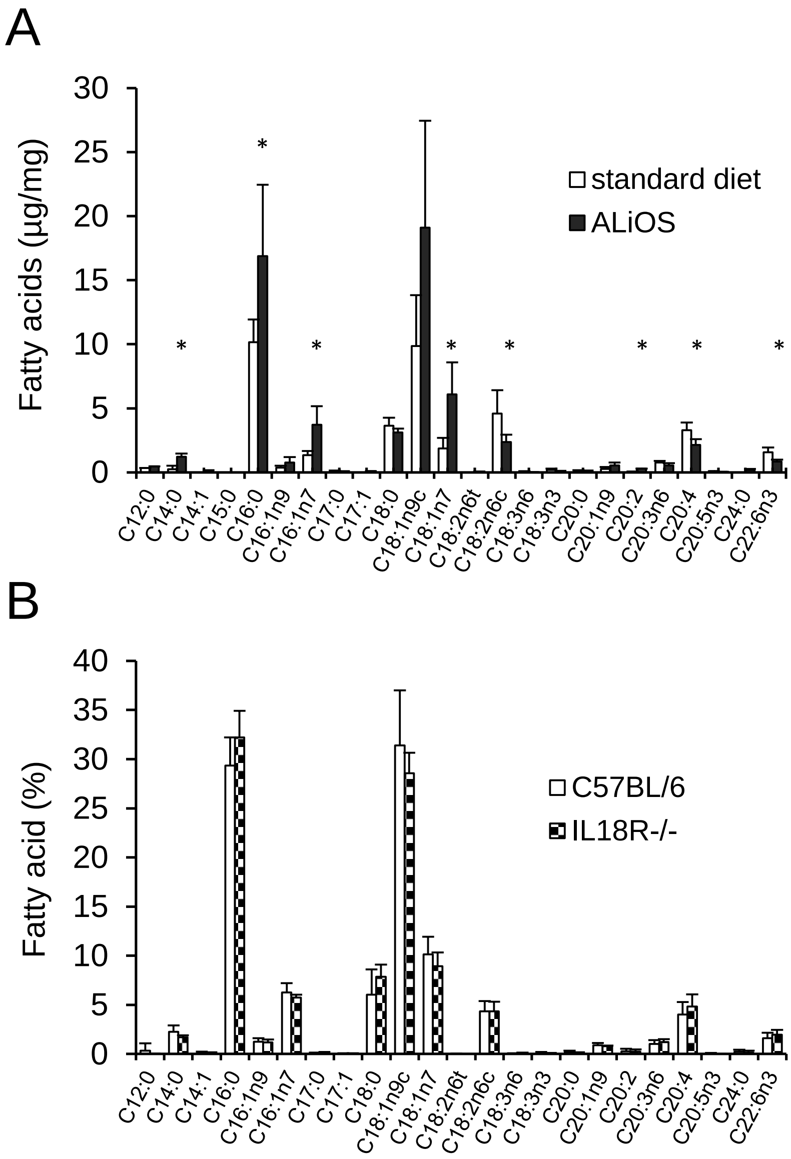

Supplementary Figure 1: Detailed lipidomic analysis in liver tissue in different diets and genotypes

(A) Wild type mice were fed standard diet (white bars) or ALiOS diet (black bars) for 24 weeks. Liver lipidome was characterized by HPLC as described and all detectable fatty acids are shown. (B) Wild type (black bars) and IL-18R-/- mice (chess field) were fed a ALiOS diet according to the ALiOS model for 24 weeks and liver lipidome is compared between the two genotypes. (mean ± standard deviation; n=5, each, *p<0.05, t-test)
